# SwissFEL Aramis beamline photon diagnostics

**DOI:** 10.1107/S1600577518005775

**Published:** 2018-06-12

**Authors:** Pavle Juranić, Jens Rehanek, Christopher A. Arrell, Claude Pradervand, Rasmus Ischebeck, Christian Erny, Peter Heimgartner, Ishkhan Gorgisyan, Vincent Thominet, Kai Tiedtke, Andrey Sorokin, Rolf Follath, Mikako Makita, Gediminas Seniutinas, Christian David, Christopher J. Milne, Henrik Lemke, Milan Radovic, Christoph P. Hauri, Luc Patthey

**Affiliations:** a SwissFEL, Paul Scherrer Institut, Villigen 5232, Switzerland; b CERN, Geneva 1211, Switzerland; c Paul Scherrer Institut, Villigen 5232, Switzerland; d DESY, Notkestrasse 85, Hamburg 22607, Germany; e Ioffe Physico-Technical Institute, Politekhnicheskaya 26, St Petersburg 194021, Russia; f European XFEL, Holzkoppel 4, Schenefeld 22869, Germany

**Keywords:** FEL physics, photon diagnostics, instrumentation, hard X-rays

## Abstract

The SwissFEL Aramis beamline, covering the photon energies between 1.77 keV and 12.7 keV, features a suite of online photon diagnostics tools to help both users and FEL operators in analysing data and optimizing experimental and beamline performance. Scientists will be able to obtain information about the flux, spectrum, position, pulse length, and arrival time jitter *versus* the experimental laser for every photon pulse, with further information about beam shape and size available through the use of destructive screens. This manuscript is an overview of the diagnostics tools available at SwissFEL and presents their design, working principles and capabilities. It also features new developments like the first implementation of a THz-streaking based temporal diagnostics for a hard X-ray FEL, capable of measuring pulse lengths to 5 fs r.m.s. or better.

## Abstract 2

The photon diagnostics available at the SwissFEL Aramis beamline are described. The working principles of various devices, their function and their expected or measured performance are discussed.

## 1. Introduction   

The rapid development of X-ray free-electron laser (XFEL) facilities like FLASH, FERMI, LCLS, SACLA, PAL-XFEL and SwissFEL (Ackermann *et al.*, 2007 ▸; Allaria *et al.*, 2010 ▸; 2013 ▸; Emma *et al.*, 2010 ▸; Ishikawa *et al.*, 2012 ▸; Oberta *et al.*, 2011 ▸; Milne *et al.*, 2017 ▸; Ko *et al.*, 2017 ▸) has brought a wave of new experiments that use the high intensities, short pulses or high coherence properties of the FEL X-ray pulses. However, both machine operators and users quickly noted that the pulse properties could and would change on a shot-to-shot basis, especially for those facilities that produced their FEL light using the self-amplified spontaneous emissions (SASE) process (Saldin & Kondratenko, 1980 ▸; Bonifacio *et al.*, 1984 ▸), making the evaluation of the data gathered during an experiment more difficult. Operators of the machines, aware of the intrinsic stochastic processes that create fluctuations in intensity, spectral distribution and pulse length, also desired some kind of monitoring mechanisms of the photon beam to optimize the machine parameters.

Though simple scintillating screens were easily adopted from the synchrotron and accelerator communities, these diagnostics devices were limited in many ways. They could only show the profile and intensity of the beam, and were typically destructive. The use of a scintillating screen to observe the photon beam meant that the end user of the beamline saw no light. On the other hand, when the screen was out, the users had no information about the beam. The need for non-destructive shot-to-shot characterization of photon pulse properties led to the development of many different photon diagnostics tools. One of the first such tools was the gas monitor detector (GMD) at FLASH, an online photon diagnostics device that measured the shot-to-shot photon beam intensity and flux (Tiedtke *et al.*, 2008 ▸).

Further developments of online photon diagnostics devices followed, with LCLS putting in basic photon diagnostics for online intensity characterization (Moeller *et al.*, 2011 ▸), installing backscattering beam position monitors (Feng *et al.*, 2011 ▸), online photon pulse spectrum monitor (Zhu *et al.*, 2012 ▸) and timing tools to characterize the FEL pulse *versus* experimental laser arrival times (Bionta *et al.*, 2011 ▸; Schorb *et al.*, 2012 ▸; Beye *et al.*, 2012 ▸) to deal with the arrival time jitter for pump–probe experiments. SACLA similarly built a series of photon diagnostics tools for position and intensity photon beam characterization (Tono *et al.*, 2013 ▸), and eventually added more diagnostics to measure the spectrum and pulse arrival time (Katayama *et al.*, 2016 ▸). Even the seeded FEL FERMI adopted some online photon diagnostics (Zangrando *et al.*, 2012 ▸), despite the higher photon pulse stability of the machine, since the devices still offered benefits for experiments and machine operation.

SwissFEL will feature improved versions of the photon diagnostics already developed and implemented at other hard X-ray SASE FELs, in addition to several new devices, such as the photon arrival and length monitor (PALM) (Gorgisyan *et al.*, 2017 ▸; Juranić, Stepanov, Ischebeck *et al.*, 2014 ▸; Juranić, Stepanov, Peier *et al.*, 2014 ▸) and the photon single-shot spectrometer (PSSS) (Karvinen *et al.*, 2012 ▸; Makita *et al.*, 2015 ▸; Rehanek *et al.*, 2017 ▸). This paper describes the devices featured at the SwissFEL Aramis beamline, which has photon energies ranging between 1.77 and 12.7 keV, and explains their working principles, measurement properties and possible applications for the users of the facility and for machine operators.

## 2. Diagnostics devices   

**Figure 1:**
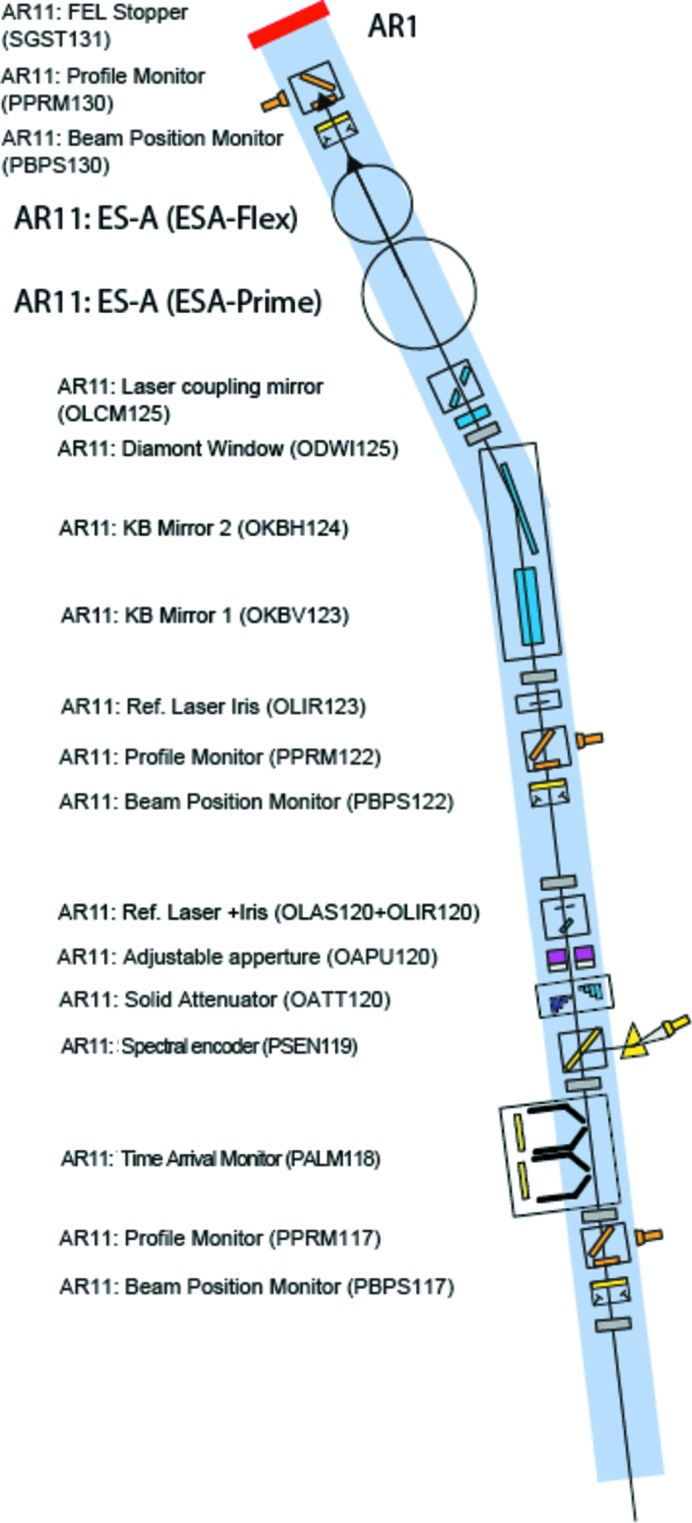
Photon diagnostics and optical component layout at end-station Alvra at SwissFEL.

The photon diagnostics at SwissFEL will provide pulse-to-pulse data on the position, flux, pulse length, spectrum and arrival time at 100 Hz, the pulse repetition rate of SwissFEL. This diagnostic data will be available in addition to the data gathered by the researchers using the experimental stations, with both being indexed by a pulse ID, allowing for quick sorting and better data analysis. Additional destructive diagnostics were built both for beam shape characterization and for preliminary spontaneous radiation studies that are required by the machine during the commissioning phase of the facility’s operation. The devices presented here are placed before the strongly focusing optical elements of the Aramis beamline, and those that insert foils or membranes into the beam do not suffer degradation or ablation due to high irradiances. The layout of the devices is shown in Figs. 1 ▸, 2 ▸, 3 ▸ and 4 ▸.

### 2.1. Gas-based photon intensity and position monitors   

**Figure 5:**
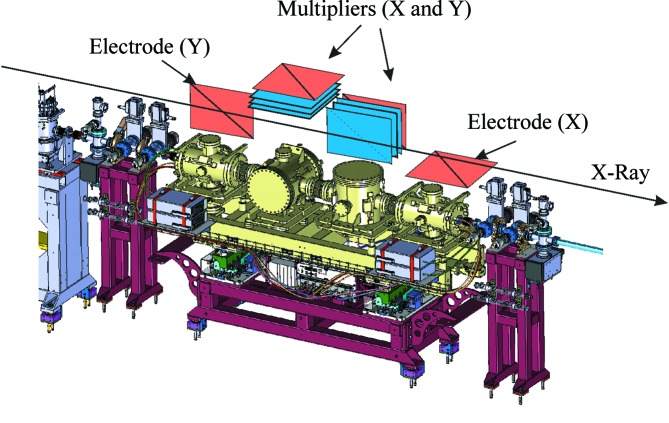
The PBIG/PBPG detector (top). The arrow indicates the direction of the FEL beam. The first and last elements are the split-electrode direct-current-measurement chambers for the vertical (upstream) and horizontal (downstream) positions of the beam and the absolute intensity. The two chambers in the middle measure the relative intensity with the help of electron multiplier plates, with the horizontal measurement upstream and the vertical downstream.

One of the most important non-destructive photon diagnostics devices at SwissFEL is the gas-based detector that measures the photon flux and the position of the beam. These two features are integrated into one device, called the photon-beam-intensity gas-monitor and the photon-beam-position gas-monitor (PBIG/PBPG). The device has grown out of developments at the DESY research institute in Hamburg for use at FLASH with soft X-rays (Tiedtke *et al.*, 2009 ▸) and has been adapted for use for the harder X-rays expected at the European XFEL facility, and underwent tests at existing hard X-ray free-electron laser facilities (Kato *et al.*, 2012 ▸; Tiedtke *et al.*, 2014 ▸). SwissFEL collaborated with the DESY photon diagnostics team to adapt the design to its needs, producing the PBIG/PBPG combination. As shown in Fig. 5 ▸, the detector consists of four separate gas-filled ionization chambers that extract the ions and electrons photoionized by the FEL beam, and tally up the charges to measure the number of atoms that were in the photoionization interaction. The gas pressure inside the device is tightly regulated and measured with a spinning rotor gauge, providing values for the gas used with an accuracy of 10% or better of the measured value over the range of gas pressures used. The gases used are noble gases, like Xe, Kr or Ar, with cross sections and mean charge state values taken from the literature (Henke *et al.*, 1993 ▸) or experiments performed by researchers from the FLASH photon diagnostics team. The photon flux loss in the device is typically below 1%, since the gas-based measurement method allows the vast majority of the photons to pass through unperturbed. Also, due to the homogeneity of the gas inside the gas detector, the wavefront of the photon pulses is not disturbed.

**Figure 6:**
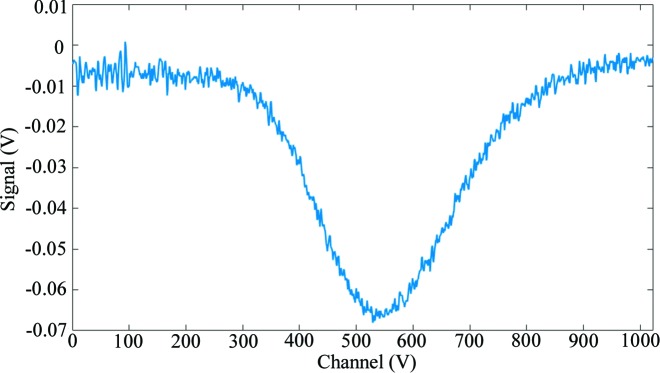
A typical single-pulse signal from one of the electrodes in the high-amplification multiplier plate (HAMP) chambers during SwissFEL commissioning, used for single-shot beam intensity and position measurement.

The two chambers at the ends of the device measure the ion current directly on split copper electrodes, while a Faraday cup collects the electrons on the other side. The ion current gives an absolute number of photons per second *N*_ph_ from the calculation in equation (1),
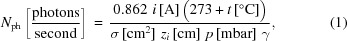
where *i* [A] is the current measured by the calibrated multimeters in amps, *t* [^o^C] is the temperature measured on the surface of the chamber in Celsius, σ [cm^2^] is the photoionization cross-section at the FEL photon wavelength in cm^2^, *z*_*i*_ [cm] is the effective length of the electrode in cm, *p* [mbar] is the pressure of the gas in mbar and γ is the average charge for the photoionized atoms at the FEL photon wavelength used. The ion current measurement is typically slow to ensure that enough charge is accumulated to yield an average current that is accurate for the absolute intensity measurements. A quicker measurement is made by Faraday cup which collects the faster-flying electrons on a pulse-to-pulse basis, and uses them to evaluate the relative strength of the photon pulse flux. The middle two high-amplification multiplier plate (HAMP) chambers also use ion signals amplified by Cu–Be multiplier plates to deliver relative shot-to-shot intensity values. These values, when combined with the slower, high-precision measurements of the ion current, yield the absolute pulse energy and photon flux for every FEL shot. The potential between the electron and ion collecting sides of the detectors can be set to as high as 20 kV to ensure that no high-energy electrons reach the ion electrodes and spoil the current measurements. Each of the chambers also measures the position of the beam in either the horizontal or vertical direction. The value *P*(*x*) = [*I*_1_(*x*) − *I*_2_(*x*)]/[*I*_1_(*x*) + *I*_2_(*x*)], where *I*_1_ and *I*_2_ are the currents of the two separate electrodes in any of the chambers, is correlated to the horizontal (*x*) position of the beam in that chamber. A similar calculation is made for the (*y*) direction in the chambers measuring the vertical position. A typical signal from the HAMP chamber used in these calculations is shown in Fig. 6 ▸.

The whole setup is mounted on a moveable frame that has the ability to shift vertically and horizontally by 3 mm in all directions in 10 µm steps. This motion of the girder is used, along with a screen behind the chambers, to calibrate the positional measurements of the detectors.

The PBIG/PBPG setup was designed to work at photon energies between 20 eV and 20 keV. The absolute flux or intensity measurement is accurate to 10% or better of the measured value, dominated by the accuracy of the gas pressure measurement, while the relative intensity measurements, dependent only on the number of generated photoions, is 1% or better. The measurement of the horizontal and vertical position of the beam is accurate to 10 µm.

### 2.2. Photon beam position monitor-solid (PBPS)   

**Figure 7:**
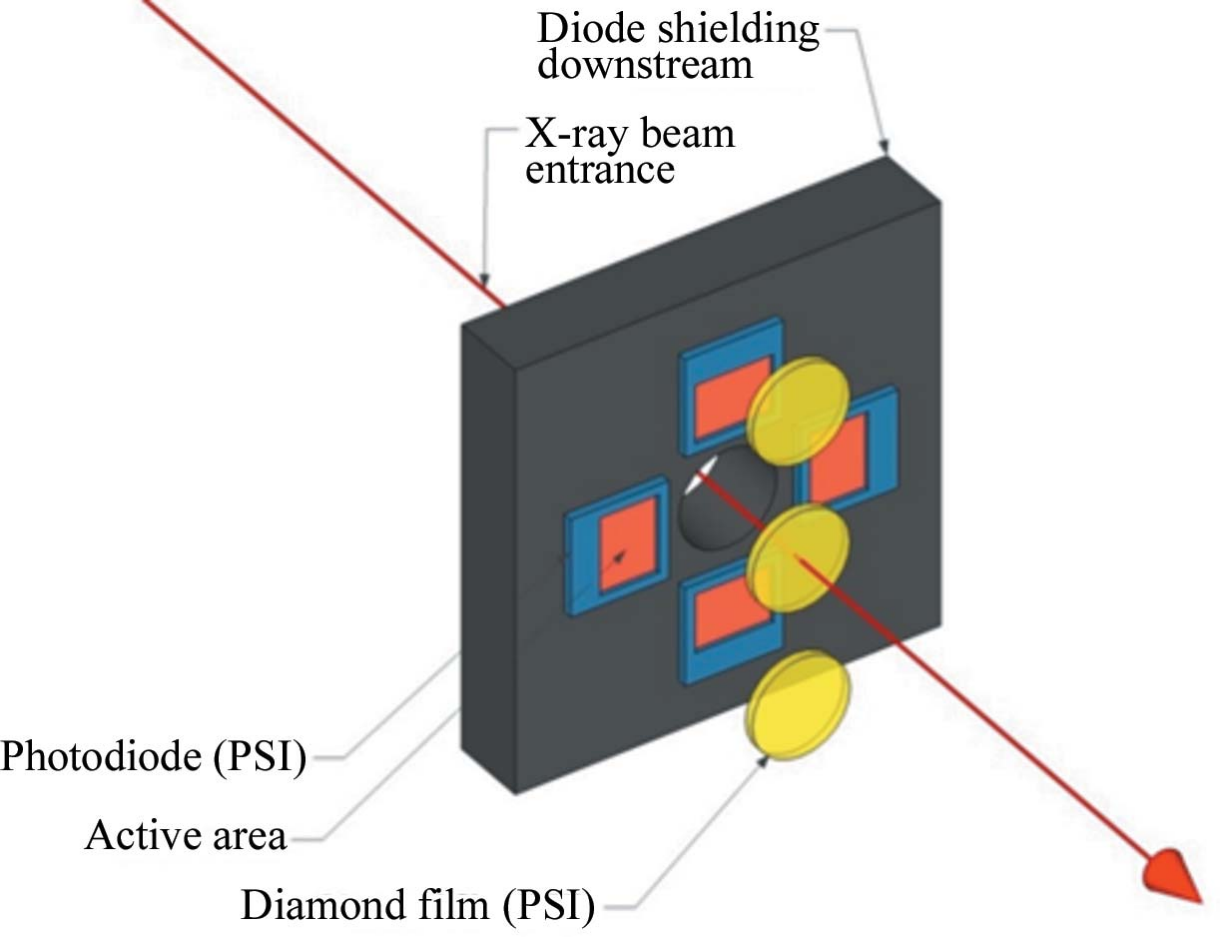
Schematic drawing of the PBPS. The plane of the diodes is about 10 mm away from the plane of the diamond discs. The area of the diamond discs visible to the light is 10 mm, and the diodes have an active surface area of 10 mm × 10 mm.

In addition to the gas detector, SwissFEL also has other types of detector to measure the intensity and position of the FEL light non-destructively. The solid photon beam position monitor (PBPS) uses a thin membrane made of diamond or Si_3_N_4_ to incoherently backscatter a portion of the photons in a pulse onto four diodes. The signal on the four diodes is then used to measure the relative pulse flux, and the absolute pulse position on a shot-to-shot basis. This design is based on the backscattering monitors developed at LCLS and SACLA (Feng *et al.*, 2011 ▸; Tono *et al.*, 2011 ▸), and has proven to be a robust and compact tool for online measurements of position and intensity for end-station users. In addition to the standard setup described above and shown in Fig. 7 ▸, the PBPS is mounted in a chamber that has the ability to be moved horizontally and vertically perpendicular to the direction of the beam by ±2 mm with 10 µm accuracy. This motion is used to calibrate the position readings of the PBPS in conjunction with a screen located downstream of the device. The PBPS is a much smaller monitor than the PBIG/PBPG that is easier to fit behind mirrors and optical components to observe the position and relative intensity of the beam as the optical parameters change. However, it cannot deliver absolute intensity values, so is used in a complementary role with the PBIG/PBPG.

The transmission of the PBPS has to be carefully balanced against the total number of back-reflected photons that are used for the position and intensity measurements. The cross-section tables from Henke *et al.* (1993 ▸) help with the former, and the latter are derived from the coherent and incoherent scattering tables for various atoms given by Hubbell *et al.* (1975 ▸) and in an integration of the backscattering photon distribution over the area covered by the diodes. Equation (2) below shows the evaluation of the incoherent backscattering effect,
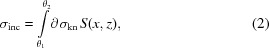
where σ_inc_ is the total incoherent cross-section per atom, *S*(*x*, *z*) is the incoherent scattering function, ∂σ_kn_ is the differential solid-angle Klein–Nishina (free-electron Compton) cross-section per electron, and θ_1_ and θ_2_ are the angles that define the cone of the photons that are being backscattered onto the diodes. The Klein–Nishina cross-section is defined by
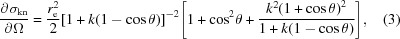
where *r*_e_ = 2.8179380 × 10^−15^ m the classic electron radius, *k* is the photon energy in units of electron rest-mass energy, eV/511003.4, and θ is the angle to the vector of the backscattered photon measured from the incoming beam.

The function *S*(*x*, *z*) is defined by a series of values for a value of *x* = sin(θ/2)/λ, where λ is the wavelength of the photon being scattered in Å. The tables of Hubbell *et al.* (1975 ▸) list the *S*(*x*, *z*) values as a function of *x* and an element like carbon or silicon. The tables also list the coherent scattering form function values, which can also calculate the coherent scattering effect of the various crystals.

Once the wavelength and the element are chosen, and the appropriate cross-section per atom has been calculated, we use a modified formula for transmission to find out how many photons are backscattered in our solid cone. The general expression for transmission *T* is
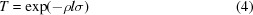
where ρ is the atomic density of the material, *l* is the thickness of the film, and σ = σ_inc_, the total incoherent cross-section per atom. The ratio of photons that are reflected would be *R* = 1−*T*. If we use the geometry displayed in Fig. 7 ▸, and described by Tono *et al.* (2011 ▸), the diodes would see photons reflected between 2.065 and 2.501 rad.

The relative intensity is then measured using the sum of the signals on all four diodes, and the position by looking at the difference in the two horizontal or vertical diodes divided by the total signal in those same diodes, with the same formula as used by the electrodes in the PBIG/PBPG. The device is designed to deliver 1% or better relative intensity measurements and beam position measurement accuracy of about 10 µm. The diode placement is designed so that the reflected light delivers at least 10^6^ photons at every wavelength to each diode.

The device can work at energies between 1.77 and 12.7 keV, though the transmission suffers unless the sample thickness is decreased at the lower energies. A commercially available Si_3_N_4_ membrane of 200 nm thickness has a transmission of about 80% at 2 keV. A 100 µm-thick disc of chemical vapor deposition (CVD) diamond has a transmission of 95% at the other end of the spectrum, at 12.7 keV. The scattering materials available for the PBPS are 200 nm-thick Si_3_N_4_, and CVD diamond discs with thicknesses of 10, 30, 50 and 100 µm. The expected ratios of the backscattered photons relative to the incoming ones for the CVD diamond discs are shown in Table 1 ▸. Every PBPS has three slots for scattering samples, and each end-station will tailor their devices with the right material and thickness for their needs.

### 2.3. Screens and destructive monitors   

Although most of the photon diagnostic components developed by SwissFEL are non-destructive and meant to be used online for shot-to-shot characterization of the X-ray pulses, some devices require the complete blockage of the beam. These devices are used for commissioning and alignment of the machine, optical components and the end-stations. They are used rarely, typically at the beginning of an experimental beam time to properly orient and adjust the experiment to the direction of the beam.

#### 2.3.1. Photon diode intensity monitor   

The first and most basic of these devices is a Hamamatsu S3590-09 Si PIN diode that can be inserted directly into the beam. The photon diode and intensity monitor (PDIM) is meant to measure the spontaneous radiation of the pre-SASE X-ray beam so that the machine operators can obtain a gain curve and align the undulators more efficiently. The device is not meant for use with a SASE beam, where the diode becomes easily saturated and useless for measurements. The accuracy of the device is completely dependent on the number of incoming photons that impact its 10 mm × 10 mm surface. Its 0.3 mm-thick depletion layer ensures that most photons that impact the diode are absorbed even at high energies. This high absorption, combined with the 3.7 eV electron–hole creation energy typical for Si PIN diodes, allows for the measurement of the photon flux on the diode’s active area to about 20% accuracy. The diode can be reverse biased to increase its working range and response time.

#### 2.3.2. Photon profile monitors   

**Figure 8:**
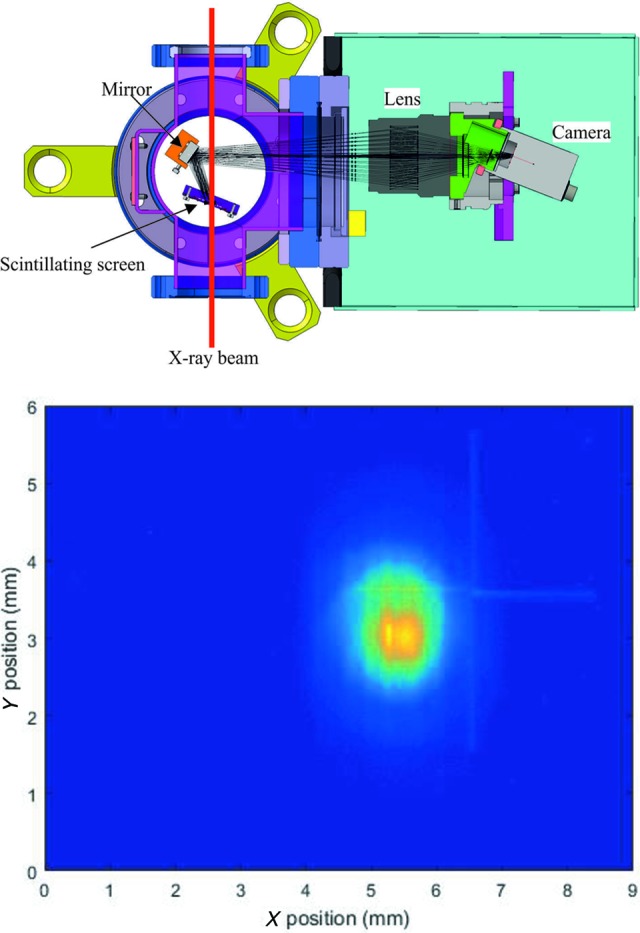
Schematic top-down view of the PPRM design with the optical paths drawn in (top) and an image taken during commissioning of one of the attenuators (bottom).

The photon profile monitor (PPRM) is a tool that uses a scintillating screen, a mirror, a lens, and a high-speed camera to acquire the photon beam images on a shot-to-shot basis. The PPRM uses a unique design developed at PSI for the transverse measurement of electron beams (Ischebeck *et al.*, 2015 ▸), using the same scintillator-and-mirror geometry and tilted camera to compensate for the Scheimpflug effect (Sheimpflug, 1904 ▸), as shown in Fig. 8 ▸, to always observe the same transverse area in the chamber. This geometry allows the insertion of several scintillators of different thicknesses into the beam path to look at the transverse photon pulse profile over a range of different photon energies and to optimize the scintillator signal and resolution. There is no way to confuse an errant motion of the motor holding the scintillator for a shift in the photon pulse position, since the mirror and the camera are always stationary and fixed relative to each other. Furthermore, the mirror is never directly exposed to the main FEL beam, ensuring that it will not lose reflectivity or become damaged with repeated use.

The PPRM was designed to provide the position and profile shape information for the alignment of the photon beam before the experimental focus to a resolution of about 11 µm. This is sufficient for the characterization of the 100 µm root-mean-square (r.m.s.) radius or bigger beam expected at SwissFEL over a 5 mm × 5 mm area in the center of the chamber. A Basler acA640-120gm 12-bit camera is used for the acquisition of the data which typically gives the transverse photon intensity measurement to an accuracy of 1% or better. The scintillators available for all PPRM are Ce:YAG crystals with thicknesses of 30, 50 and 100 µm, any of which can be put into the beam path for any of the PPRMs. As mentioned in the previous sections, the PPRMs will also be used in conjunction with online position-measuring devices to calibrate their readings.

#### 2.3.3. Photon screens for optics   

**Figure 9:**
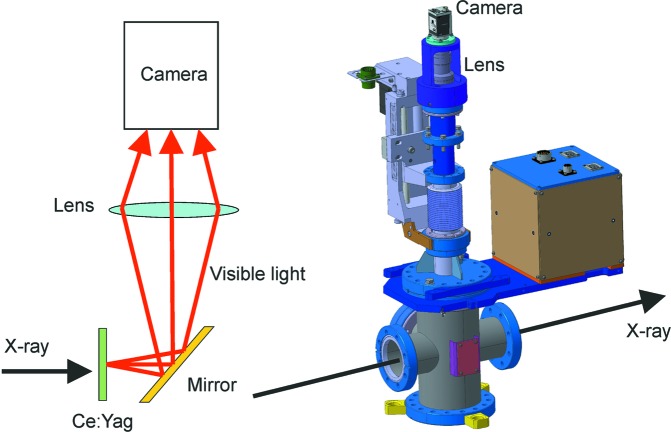
Schematic side view of the PSCR design and a three-dimensional drawing of the device.

While the PPRM is a screen system designed to look at a photon beam that moves with a fairly small jitter in a defined area, the photon screen for optics (PSCR) is a screen made to move with the beam for alignment of mirrors and monochromators. The simple design, shown in Fig. 9 ▸, allows for the camera to stay focused on the scintillating screen while the whole apparatus moves up and down. This property allows the screen to follow the beam as it is adjusted by the mirror or monochromator elements over a 50 mm distance, speeding up the process of threading the photon beam through all the optics to the end-stations during the setup and commissioning phase of the SwissFEL project. Because the PSCR is concerned only with finding the rough position of the beam and following it, the PSCR offers a resolution of only about 50 µm. The camera type used to take pictures is the same as for the PPRM.

#### 2.3.4. Photon spontaneous radiation monitor   

**Figure 10:**
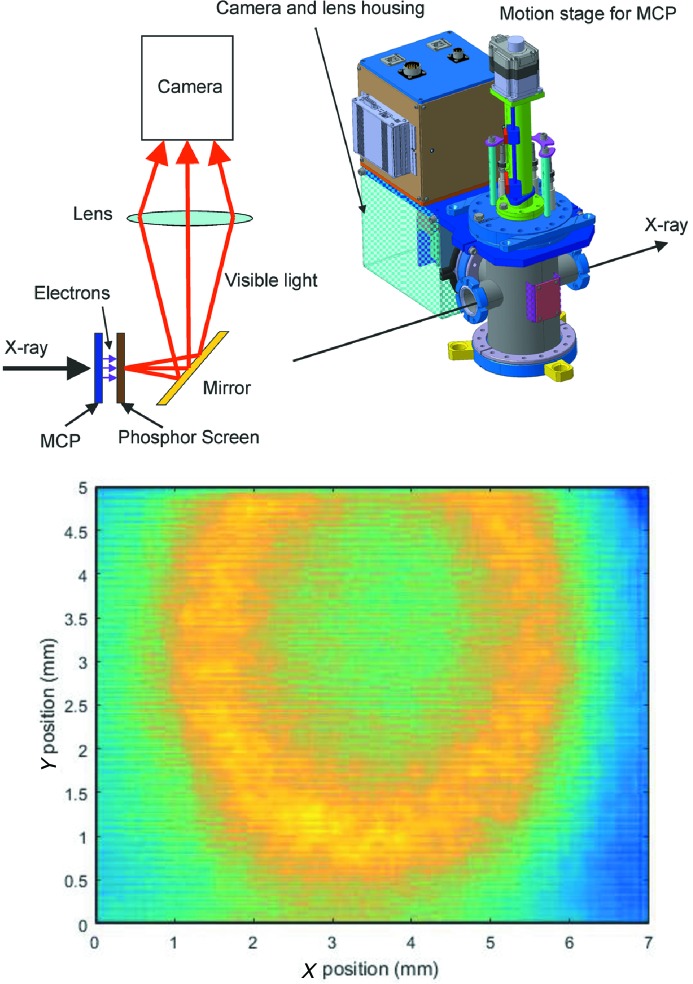
Schematic view of the PSRD (top), and a measurement taken of third-harmonic spontaneous radiation from the FEL through the monochromator during undulator commissioning (bottom).

In addition to the commissioning of optics elements, the machine operators require photon diagnostics and a monochromator to align the undulators for optimum operation (Tanaka *et al.*, 2012 ▸). The device chosen to help with this task is the photon spontaneous radiation monitor (PSRD), a multi-channel plate (MCP) mounted in front of a phospho­rous screen. A mirror mounted at 45° to the propagation of the beam reflects the light from the screen through a viewport of the chamber, where it is then recorded by a camera at 100 Hz frame rate. The machine operators can use spontaneous radiation from the undulators, and change the undulator parameters to scan the photon energy across the monochromator bandwidth to find the optimal radiation settings set by observing the shape and intensity of the recorded spontaneous radiation. This process can be used to match the position, gap size and phase of the undulators, and also correct issues such as electron trajectory problems. The PSRD was designed to give an intensity resolution of about ±1% for spontaneous radiation, and have a position resolution of about 25 µm r.m.s. The position resolution is heavily dominated by the size of the electron shower that the MCP produces after the impact by the photons and the subsequent spread of the electrons as they are accelerated to the phosphor screen behind it. The use of multiple MCPs would make the resolution worse, but additional MCPs can be added to the setup in case the machine operators wish to sacrifice resolution for higher gain for their measurements. The PSRD is schematically shown in Fig. 10 ▸. The PSRD is used in both single-shot and averaged-shot mode, depending on the intensity of the spontaneous radiation used, as many of the measurements used to align the undulators work best with little light, where the slightest optimization increases the signal. Typically, the PSRD can work in single-shot mode at photon intensities that are of the order of 10^6^–10^7^ photons per pulse.

### 2.4. Temporal diagnostics   

One of the main new scientific techniques that profited enormously from the development of FELs is that of pump–probe spectroscopy. The short pulses, high intensities and harder X-ray energies available at an FEL, when combined with a synchronized experimental IR or THz source, allow for a range of new experiments that could not be achieved by any other source before. However, as the technique has developed further, the demand for measurements with better time resolution became evident. All FELs have a residual laser-to-FEL timing jitter (Divall *et al.*, 2014 ▸). This is related due to a stochastic process in the acceleration of the electron bunch. The relative timing jitter and absolute stability of each LINAC module, including the corresponding subcomponents like klystrons and modulator, lead to an intrinsic timing jitter of the electron bunch along the several hundreds of metres long electron accelerator. Furthermore, for unseeded FELs, the stochastic SASE process leads to a jitter between the X-ray pulse and the electrons, as well as the FEL-pulse length. While the first source can be controlled by improved timing systems and better overall stability of the individual components, the second is in the nature of the SASE process and will always exist. FEL scientists have been attempting to come up with systems to non-invasively characterize both pulse length and arrival time changes of the pulses on a shot-to-shot basis. This information is used to properly analyse the data collected by experimental users, while the machine operators can use the temporal measurements to correct the performance of the machine, or correct drifts.

#### 2.4.1. Pulse arrival and length monitor   

The idea of using a THz streak camera to measure the pulse length of an FEL beam was first proposed and demonstrated at the soft X-ray FLASH FEL with the use of a special undulator as a THz source (Fruhling *et al.*, 2009 ▸; 2011 ▸). Further tests at FLASH by different groups have also shown that the technique can use a branch of the experimental laser to generate the THz beam. This development enabled the use of the THz streak camera to measure the arrival time of the FEL pulse relative to the laser beam and the pulse length of the FEL at the same time at FLASH and at the soft X-ray beamline at LCLS (Grguras *et al.*, 2012 ▸; Helml *et al.*, 2014 ▸). A dedicated THz streaking device called the pulse arrival and length monitor (PALM) was developed at PSI (Juranić, Stepanov, Peier *et al.*, 2014 ▸) and tested, first at a laser high-harmonic-generation (HHG) source (Ardana-Lamas *et al.*, 2016 ▸) and then with hard X-ray FEL pulses at SACLA (Gorgisyan *et al.*, 2017 ▸; Juranić, Stepanov, Ischebeck *et al.*, 2014 ▸), where it also proved capable of measuring double pulses at different photon energies in a special FEL mode (Hara *et al.*, 2013 ▸). The PALM is, thus far, the only example of a dedicated THz-streak based timing tool at any FEL.

The theory behind the THz streak camera and the PALM has been presented in other papers (Gorgisyan *et al.*, 2017 ▸; 2016 ▸; Juranić, Stepanov, Ischebeck *et al.*, 2014 ▸; Juranić, Stepanov, Peier *et al.*, 2014 ▸). The device uses a lithium niobate crystal and the tilted wavefront method to generate a THz pulse that co-propagates with the FEL pulse to an interaction region where the FEL pulse photoionizes a gas. The photoelectrons from this process change their kinetic energy depending on the time of the photoionization by the FEL pulse relative to the THz pulse. The photoionized electrons interact with the THz vector potential, which itself changes over time. The change in photoelectron energy is measured by electron time-of-flight spectrometers (eTOFs), encoding the femtosecond-level jitter in the arrival time between the FEL and laser/THz pulses to a nanosecond-level shift in the electron flight times. This method is also sensitive to FEL pulse length variations, as the difference between the energy gained at the head and tail of an FEL pulse from the vector potential stretches the photoelectron peak, making it broader. The streaked and non-streaked spectra are measured simultaneously at two separate locations and compared directly with each other to extract the pulse length and arrival time.

The PALM setup tested at SACLA could measure the arrival time accuracy as accurately as 4 fs r.m.s. Its pulse length measurements at the HHG source have proven to be accurate down to a pulse length of 25 fs r.m.s. with a top accuracy of 2 fs r.m.s. The evaluations of the pulse length and accuracies are heavily dependent on the condition of the FEL beam. However, the PALM has proven to be a reliable tool for most beam modes used at an FEL facility, including the measurement of the photon pulse and arrival time with a monochromator, something other temporal diagnostics methods have issues with (Juranić, Stepanov, Ischebeck *et al.*, 2014 ▸). However, the THz streaking method is limited to a range of arrival times of about 600 fs.

**Figure 11:**
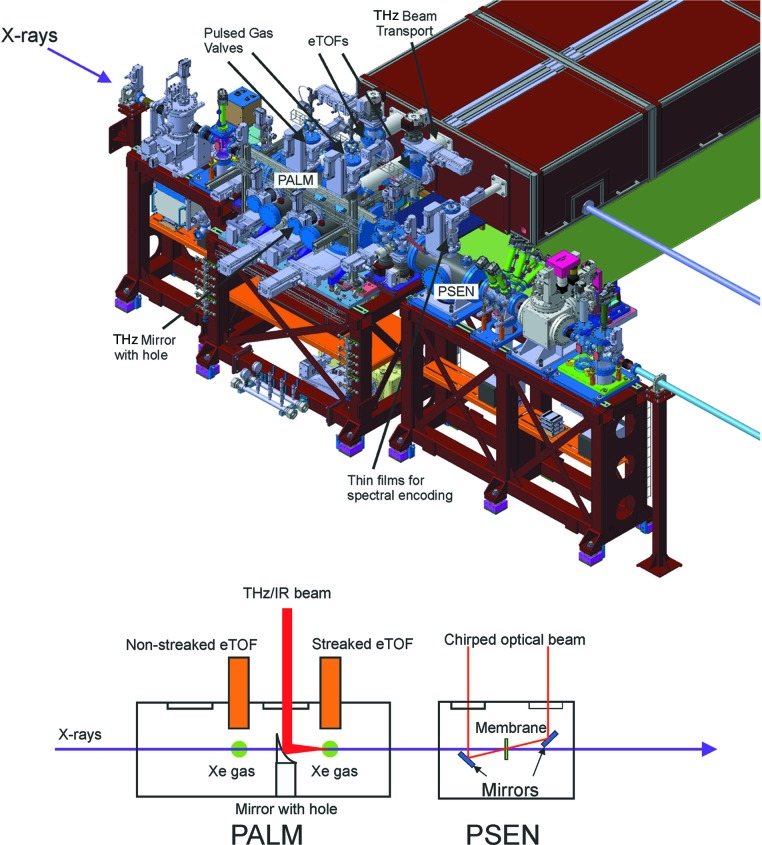
Schematic overview of the PALM and the PSEN at one of the SwissFEL end-stations.

The PALM setup at PSI has a 10 mJ dedicated laser source for THz generation, branched off from the main experimental laser to maximize the synchronization between the diagnostic and experimental measurements (Erny & Hauri, 2016 ▸). The device also consists of alignment optics, electro-optical (EO) sampling crystals and a high-bandwidth diode. The EO sampling crystal is used to find the temporal and spatial overlap between a probe IR beam and the THz pulse in the interaction region, and the diode is used to find the overlap between the probe IR beam and the FEL. With these two parameters measured, the THz and FEL can be easily overlapped in space and time by adjusting out-of-vacuum laser optics and changing the flight path of the THz-generating laser beam *via* a moveable stage. Once set up, the device takes a streaked and non-streaked spectrum for every pulse, and directly compares them, providing the users with pulse length and arrival time information along with the collected data. A set of pulsed gas valves from MassSpecpecD are synchronized to inject gas for 15–30 µs into the chamber right before the photon pulse arrives, while Kaesdorf ETF-20b eTOFs are positioned to measure the photoionized electron spectra at both the upstream (non-streaked) and downstream (streaked) positions. The eTOFs are positioned along the plane of the FEL polarization to observe the maximum electron signal with regards to the electron angular distribution from ionization. The THz polarization is set to match the FEL polarization, maximizing the streaking effect. The THz radiation is created on the diagnostic laser table and coupled into the PALM chamber through a special z-cut quartz window. The in-vacuum THz mirror focuses the THz beam onto the downstream interaction region, while a 3 mm hole in its middle allows the FEL beam to pass through undisturbed. The mirror’s position can be adjusted to optimize the focus or to remove it completely from the beam. A drawing of the temporal diagnostic section in Fig. 11 ▸ shows the overall setup. As with the gas detector, the gas-based nature of the PALM means that typically 1% or less of the incoming light is used for the temporal measurement, and it does not disturb the wavefront.

#### 2.4.2. Photon spectral encoder   

A photon spectral encoder (PSEN) is also available for user operation. Spatial and spectral encoding devices have been successfully used at other FEL facilities like LCLS and SACLA (Bionta *et al.*, 2011 ▸; Katayama *et al.*, 2016 ▸), and have proven their ability to measure arrival time to an accuracy of several femtoseconds (Harmand *et al.*, 2013 ▸; Katayama *et al.*, 2016 ▸). Cross-calibration experiments at SACLA have shown that the measurements with these types of devices agree very well with measurements from the PALM (Gorgisyan *et al.*, 2017 ▸). The PSEN and the PALM are meant to work together to cross-check and cross-calibrate each other’s arrival time measurements.

The principle of spectral encoding and the PSEN works by chirping an optical laser pulse over a length of several picoseconds. Here the chirp in wavelength directly correlates to the chirp in time so that each wavelength comes at a distinct time. The chirped pulse is then sent through a thin dielectric film, such as Si_3_N_4_. When the FEL beam interacts with the dielectric, it changes its optical properties, resulting in a change in transmission. If the chirped optical pulse is passing through the thin film at the same time, its transmitted intensity will change. The optical laser is analysed by a spectrometer, and the arrival time of the FEL pulse is obtained from the change in transmission of the wavelength of the chirped optical pulse. To ensure that the optical transition contrast is most visible, it is important that the optical laser beam is focused on the dielectric thin film to a spot that is smaller than the FEL pulse spot size. At the SwissFEL, this means that the optical laser is focused to a size no greater than 150 µm.

The main concern when using a PSEN-like device is the invasiveness of the measurement. The change in transmission of the optical laser needs to be large enough to be analysed, which requires that many of the FEL pulse’s photons interact with the material to change its properties, leading to the use of thicker dielectric films. However, these films would absorb too many photons, and the device would be invasive for the users at the end of the beamline. To deal with this issue, SwissFEL can use several different thin films of various thicknesses, from 100 nm-thick Si_3_N_4_ to 10 µm-thick Ce:YAG.

However, even with these precautions, the PSEN would have difficulty measuring the arrival time completely non-invasively when the FEL photons are limited, as is the case when the experiments use a monochromator. The PALM can step in and take up the slack, while the PSEN will be invaluable for experiments requiring the measurement of arrival times over large jitter or delays, since it can work quite well over a range of several picoseconds, something that the PALM cannot easily do. The two measurements method can be combined together to increase the pulse length and arrival time resolution and performance of both with the method described by Juranić, Stepanov, Peier *et al.* (2014 ▸).

### 2.5. Photon single-shot spectrometer   

The measurement of the photon spectrum on a shot-to-shot basis has become a very important part of most proposed FEL experiments. In addition to the experimental need to compensate for the spectral jitter caused by the intrinsic stochasticity in the SASE process, experiments are being proposed that require new special spectral modes that an online spectrometer could measure. Experiments requiring a large bandwidth mode or those that wish to look at speckle measurements from photon–sample interactions must be able to differentiate between pulses that were generated using light that is suitable for the experiment and false results that may be occurring due to some change in the photon beam. The photon single-shot spectrometer (PSSS) was developed to deliver pulse-to-pulse photon spectrum measurements.

The PSSS combines the bent crystal spectrometer, such as the one developed at LCLS (Zhu *et al.*, 2012 ▸), with a grating that separates a small portion of the light from the zeroth order that proceeds downstream to the experiments (Karvinen *et al.*, 2012 ▸), similar to the design at SACLA (Katayama *et al.*, 2016 ▸). The grating is made of diamond to increase the transmission of the light to downstream experiments and reduce the possibility of damage to the grating due to photon absorption. The separated first-order diffracted light is steered onto a bent crystal, and then used to measure the photon spectrum of every FEL pulse. The first experiments performed at LCLS (Makita *et al.*, 2015 ▸), led by a group from PSI who manufactured the diamond gratings (Makita, 2017 ▸) for the beam separation, performed a proof of principle, and the development of the PSSS started shortly after. The full design, test and construction considerations of the PSSS are given by Rehanek *et al.* (2017 ▸).

**Figure 12:**
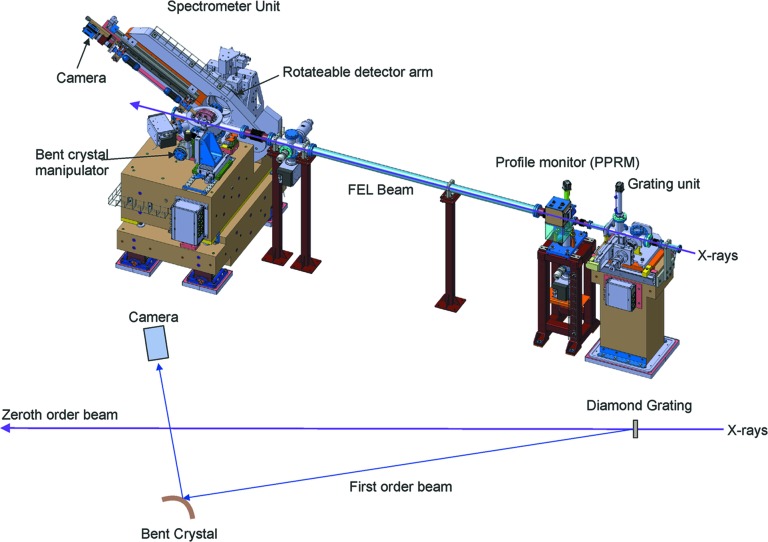
Schematic overview of the PSSS.

As shown in Fig. 12 ▸, the FEL light is split in the grating chamber, typically with 90% or more intensity of the beam in the zeroth order being transmitted to the end users further downstream. The first-order beam typically carries 0.2–1% of the incoming radiation, and is diffracted towards the spectrometer chamber, located about 4 m downstream of the grating chamber. This beam is then Bragg-reflected from bent crystals, and then dispersed onto a scintillating screen positioned at the end of a meter-long helium-filled tube. A fast PCO Edge camera with an objective records the spectra and delivers them to the data acquisition and analysis system. The spectral profile of every pulse is recorded, and can be bundled with the data that users and operators receive for every shot. There is also a screen between the grating chamber and the spectrometer chamber to measure the spatial profile of the deflected pulse. This recording is used to compensate for the spatial intensity distribution in the final spectrum. The system also delivers a center-of-mass energy measurement for spectral stability measurements.

The grating chamber has the capability to insert diamond gratings with a pitch of 100, 150 and 200 nm into the beam. An appropriate choice of grating pitch and X-ray energy can separate the first and zeroth order from each other by an angle of typically 1 mrad, which is sufficient to allow the downstream spectrometer unit to use the first-order beam without disturbing the zeroth order. The manipulators in the chamber can also tilt the gratings by up to 60° to increase the effective depth of the gratings and increase the diffraction efficiency. The spectrometer unit contains slots for four bent 10 µm-thick Si crystals, currently consisting of a Si (111) crystal with a bending radius of 155 mm, and three Si (220) crystals with bending radii of 75, 145 and 200 mm. The spectrometer camera is mounted on a rotating arm that allows it to follow the Bragg angle as the photon energy changes. The combination of the grating, bent crystals and rotating detector arm allows the PSSS to measure photon energies between 4 and 12.7 keV. The relative resolution Δ*E*/*E* is between 2 × 10^−5^ and 5 × 10^−5^ over a bandwidth of 0.5% of the photon energy. Lower X-ray energies are difficult to measure since X-ray absorption by the diamond increases, and the photon–material interactions become intense enough that they can potentially degrade the diamond gratings to a point that they no longer diffract the beam.

## 3. Conclusion and overview   

The photon diagnostics devices developed for SwissFEL have been built to anticipate the demands of both users and machine operators, and will provide access to the non-destructive characterization of XFEL photon flux, position, spectrum, pulse length and arrival time. The machine, beamline and experimental scientists will be able to use the various screen and diode-based invasive and non-invasive devices to commission the beamline and machine components, and also align the optics to optimize the FEL performance. The devices will be fully integrated into the SwissFEL control system, and deliver the measured data to the users and operators on a shot-to-shot basis for fast analysis. The goal is to provide immediate feedback that allows for fast and accurate evaluation of the performance of the beamline or the status of the machine, so that an appropriate decision for the next step in the experiments can be made quickly. The novelty in the presented setup comes from the integration of the diagnostics into the experimental systems from the design stage, and the first use of THz streaking as a standard shot-to-shot analysis tool for temporal diagnostics. Every device used has been extensively tested and optimized, ensuring that the performance given at SwissFEL is the best possible.

## Figures and Tables

**Figure 2:**
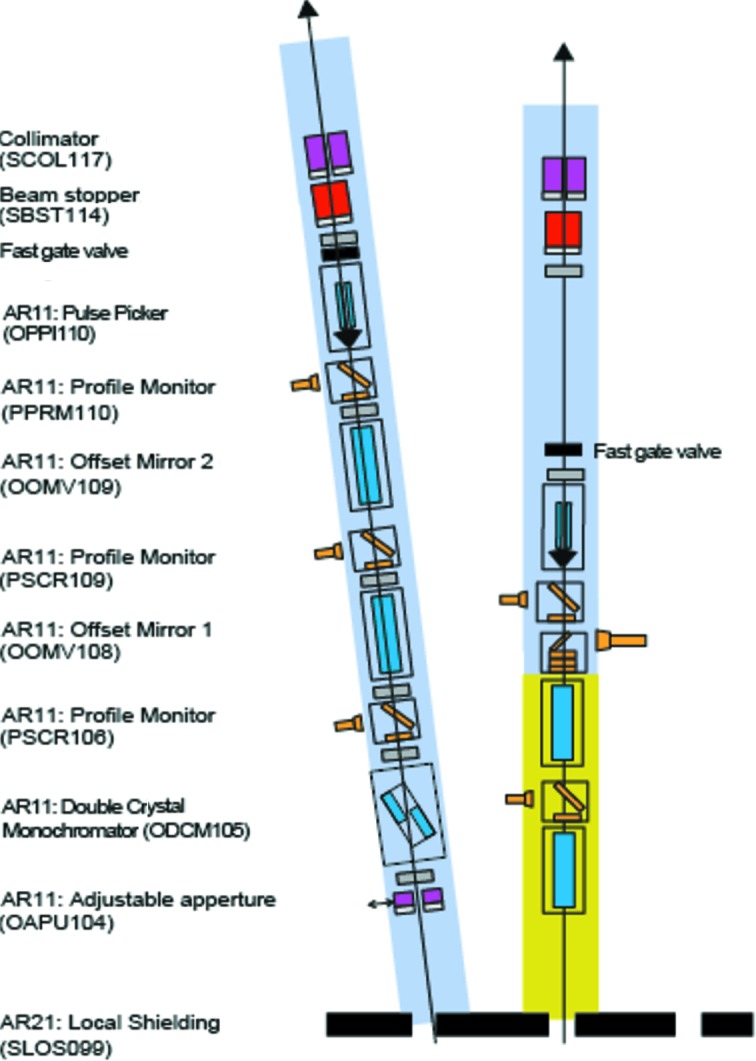
Photon diagnostics and optical layout in the downstream part of the optics hutch of SwissFEL.

**Figure 3:**
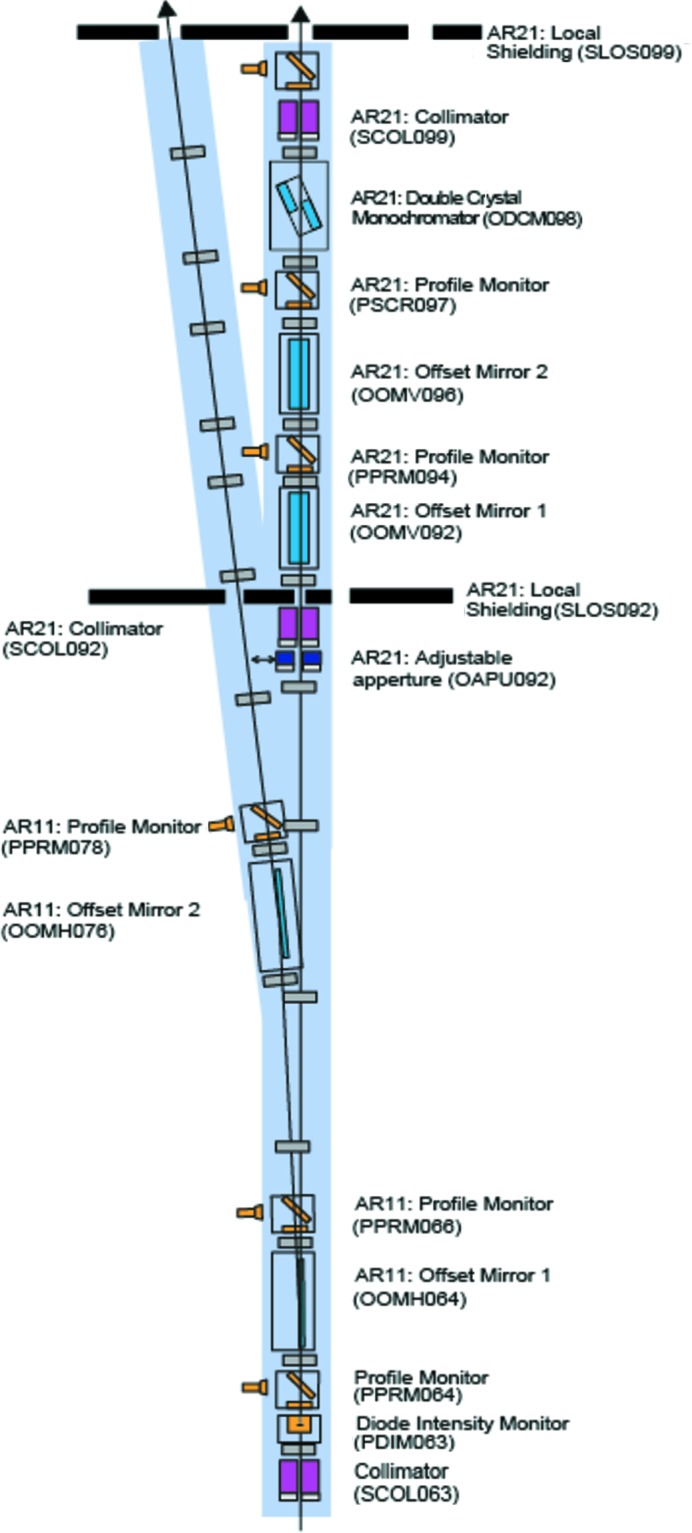
Photon diagnostics and optical layout in the upstream part of the optics hutch of SwissFEL.

**Figure 4:**
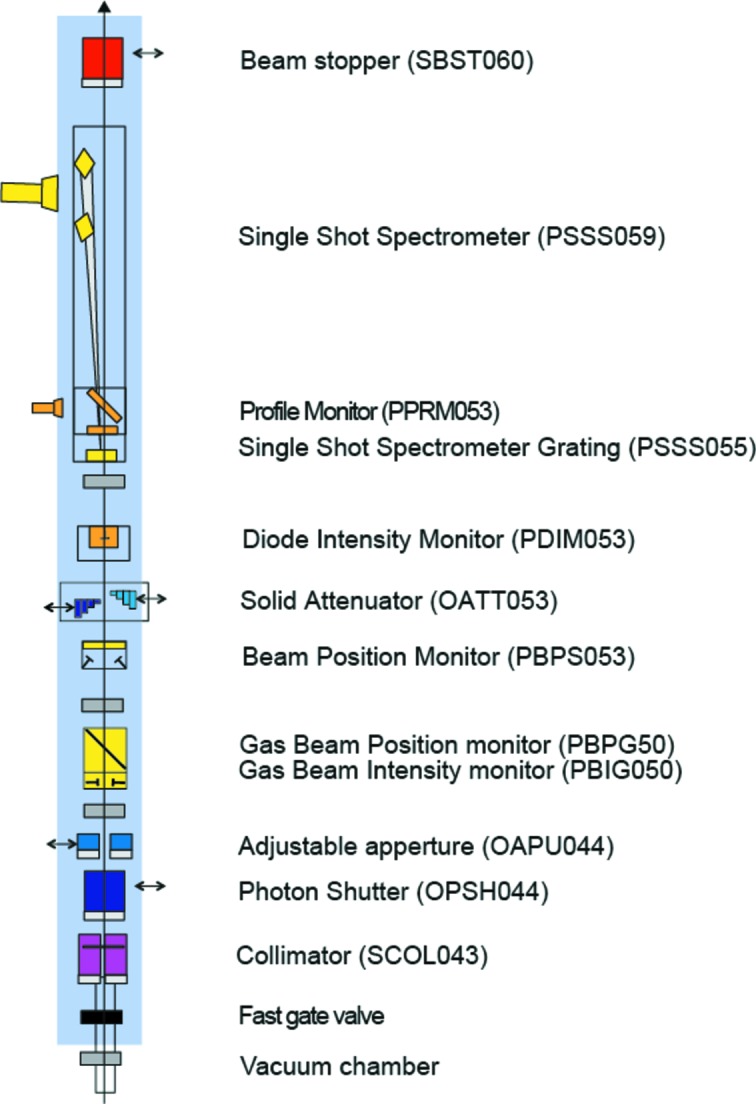
Photon diagnostics and optical layout in the front-end of SwissFEL.

**Table 1:** The estimated portion of the incoherent reflected photons, for the PBPS geometry shown in Fig. 7 ▸, that impact the four diodes for different energies and diamond disc thicknesses

	Estimated reflected photon ratio for CVD diamond discs per diode			
Photonenergy (eV)	10 µm	30 µm	50 µm	100 µm
4130	9.5 × 10^−6^	2.8 × 10^−5^	4.7 × 10^−5^	9.5 × 10^−5^
6200	1.6 × 10^−5^	4.8 × 10^−5^	8.1 × 10^−5^	1.6 × 10^−4^
12400	2.1 × 10^−5^	6.5 × 10^−5^	1.1 × 10^−4^	2.1 × 10^−4^
