# SwissFEL Aramis beamline photon diagnostics. Erratum

**DOI:** 10.1107/S1600577519005654

**Published:** 2019-05-01

**Authors:** Pavle Juranić, Jens Rehanek, Christopher A. Arrell, Claude Pradervand, Ariana Cassar, Marco Calvi, Rasmus Ischebeck, Christian Erny, Peter Heimgartner, Ishkhan Gorgisyan, Vincent Thominet, Kai Tiedtke, Andrey Sorokin, Rolf Follath, Mikako Makita, Gediminas Seniutinas, Christian David, Christopher J. Milne, Henrik Lemke, Milan Radovic, Christoph P. Hauri, Luc Patthey

**Affiliations:** a SwissFEL, Paul Scherrer Institut, Villigen 5232, Switzerland; b Department of Microelectronics and Nanoelectronics, University of Malta, Msida, Malta; c Paul Scherrer Institut, Villigen 5232, Switzerland; d CERN, Geneva 1211, Switzerland; e DESY, Notkestrasse 85, Hamburg 22607, Germany; f Ioffe Physico-Technical Institute, Politekhnicheskaya 26, St Petersburg 194021, Russia; g European XFEL, Holzkoppel 4, Schenefeld 22869, Germany

**Keywords:** FEL physics, photon diagnostics, instrumentation, hard X-rays

## Abstract

The list of authors in the paper by Juranić *et al.* (2018) [*J. Synchrotron Rad.***25**, 1238–1248] is corrected.

Two authors, Ariana Cassar and Marco Calvi, were omitted in the published version of the paper by Juranić *et al.* (2018). The correct author list is given above.
